# Editorial: Ocular fibrosis: molecular and cellular mechanisms and treatment modalities

**DOI:** 10.3389/fopht.2024.1362601

**Published:** 2024-01-25

**Authors:** Christine M. Sorenson, Teri L. Belecky-Adams, Nader Sheibani

**Affiliations:** ^1^ Department of Pediatrics, University of Wisconsin School of Medicine and Public Health, Madison, WI, United States; ^2^ McPherson Eye Research Institute, University of Wisconsin School of Medicine and Public Health, Madison, WI, United States; ^3^ Department of Biology, Indiana University-Indianapolis, Indianapolis, IN, United States; ^4^ Department of Ophthalmology and Visual Sciences, University of Wisconsin School of Medicine and Public Health, Madison, WI, United States

**Keywords:** ocular fibrosis, myofibroblasts, extracellular matrix, inflammation, microRNA, integrins

Tissue fibrosis occurs as an excessive repair response to mechanical damage, inflammation, ischemia, and degeneration, leading to irreversible scar formation (1). The excessive proliferation and deposition of various extracellular matrix (ECM) proteins are mediated through the transition of various cell types to myofibroblast-like cells. These cell types include epithelial cells, fibroblasts, vascular cells, glial cells, and inflammatory cells (2). The noted phenotypic and functional changes are considered major events during the fibrotic process with severe alterations in the tissue integrity and function (3, 4). Fibrosis contributes to the severity of pathologies in many chronic inflammatory diseases in the eye including retinopathy of prematurity, proliferative diabetic retinopathy (PDR), age-related macular degeneration (AMD), and glaucoma (1, 5). Although the mechanisms of fibrogenesis have been extensively studied, the exact molecular and cellular events that drive this destructive tissue insult remain unresolved and may vary in a tissue, cell, and context-dependent manner. Despite great advances in our knowledge regarding its etiology, effective treatment of fibrogenesis presents an unmet challenge.

Numerous *in vitro* and *in vivo* models of fibrogenesis have helped gain insight into the role of various factors and their impact on intracellular mechanisms that drive the changes associated with fibrotic responses. Unfortunately, targeting many of these cells and pathways has proven ineffective in the prevention and reversal of fibrotic responses in various diseases. Numerous growth factors including TGF-β, PDGF, CTGF, VEGF-A, and TNF-α have profibrotic activities and contribute to ocular fibrosis through the activation of their downstream signaling events (6). However, the unique contribution of these pathways and the target cells involved suggests a multi-step process whose involvement in different steps needs further investigation. Although myofibroblasts are recognized as key cell types contributing to fibrosis, their sources remain unclear and may involve specific cell types in a unique tissue, cell, and organ-specific manner. Thus, the investigation of tissue and cell-specific mechanisms is vital to the identification of common and distinct contributors to fibrosis. This knowledge should aid in the development of more effective strategies to halt and/or reverse fibrotic responses associated with various chronic diseases.

Recent studies identifying the source of myofibroblasts, the key cell type driving fibrogenesis, have led to the identification of perivascular supporting cells in many tissues in response to damage including diabetic retinopathy and age-related macular degeneration (7–9). However, the details of mechanisms for this selective transition of pericytes to myofibroblasts and the source of mediators involved in fibrogenesis need further investigation. One of the key pathways implicated in fibrogenesis involves increased production of TGF-β and its action through its canonical (Smad-dependent) and non-canonical (Smad-independent) pathways driving fibrosis.

TGF-β is shown to impact the transition of various cell types including epithelial cells, vascular cells, and inflammatory cells to myofibroblast-like cells and enhance the production of various ECM proteins. These changes have a significant impact on the ECM milieu and the function and integrity of the tissue and cellular microenvironment. Retinal pigment epithelium (RPE) cell dysfunction contributes to many retinal diseases including PDR, vitreoretinopathy, macular damage, and retinal detachment (10). Higashijima et al. review the important role of TGF-β in promoting the transition of RPE cells to myofibroblast-like cells and the mediation of subretinal fibrosis associated with neovascular AMD. Zhao et al. (11) showed that TGF-β can also mediate pericyte to myofibroblast transition in subretinal fibrosis. Thus, the significant contribution of these different cellular transitions to subretinal fibrogenesis deserves further investigation.

Recent studies have also investigated the potential regulatory role microRNAs may play in mechanisms that promote the transition to myofibroblast. Leng et al. show that miRNA-494 promotes RPE cell transitions to myofibroblasts by targeting P27, which is an important regulator of cell proliferation. In addition, miRNA-494 may similarly promote the transition of Müller cells to myofibroblast, contributing to the formation of the epiretinal membrane. These studies provide additional insight into potential regulatory mechanisms that impact the transition to fibrogenesis.

Given the significant role of inflammatory processes in fibrogenesis, the use of anti-inflammatory agents could prevent fibrogenesis and provide protection. Wong et al. show that a liposomal steroid delivery is effective in mitigating fibrosis in a preclinical model of proliferative retinopathy. Inflammatory mediators also play a significant role in the modulation of ECM proteins involved in the remodeling of tissue microenvironment, which favors fibrogenesis. Many actions of ECM proteins are mediated through their interactions with cell surface receptors called integrins. Faralli et al. provide a detailed overview of various integrins expressed in the trabecular meshwork and their potential involvement in fibrogenesis changes impacting outflow facility, and as a result, the intraocular pressure and optic nerve damage in glaucoma.

Although these studies aid in our understanding of the cellular and molecular mechanisms that drive fibrogenesis, additional investigations are needed to further identify the target cells and pathways that drive their fibrogenic phenotype in a tissue and cell-specific manner. Advances in RNA sequencing have begun to shed light on these questions, identifying cells with unique fibrotic properties and identifying key mediators that drive the transition process and ECM changes (9, 12, 13). This knowledge will aid in tissue and cell-specific targeted strategies to effectively prevent and reverse fibrosis.

## Author contributions

CS: Writing – original draft, Writing – review & editing. TB: Writing – original draft, Writing – review & editing. NS: Writing – original draft, Writing – review & editing.

## References

[1] Mallone F Costi R Marenco M Plateroti R Minni A Attanasio G. Understanding drivers of ocular fibrosis: current and future therapeutic perspectives. Int J Mol Sci (2021) 22(21):11748. doi: 10.3390/ijms222111748 34769176 PMC8584003

[2] Greaves NS Ashcroft KJ Baguneid M Bayat A. Current understanding of molecular and cellular mechanisms in fibroplasia and angiogenesis during acute wound healing. J Dermatol Sci (2013) 72(3):206–17. doi: 10.1016/j.jdermsci.2013.07.008 23958517

[3] Abu El-Asrar AM Nawaz MI Ahmad A Siddiquei MM Allegaert E Gikandi PW. CD146/soluble CD146 pathway is a novel biomarker of angiogenesis and inflammation in proliferative diabetic retinopathy. Invest Ophthalmol Vis Sci (2021) 62(9):32. doi: 10.1167/iovs.62.9.32 PMC8300056 34293080

[4] Chaudhary R Scott RAH Wallace G Berry M Logan A Blanch RJ. Inflammatory and fibrogenic factors in proliferative vitreoretinopathy development. Transl Vis Sci Technol (2020) 9(3):23. doi: 10.1167/tvst.9.3.23 PMC7357815 32742753

[5] Hachana S Larrivée B. TGF-β Superfamily signaling in the eye: implications for ocular pathologies. Cells (2022) 11(15):2336. doi: 10.3390/cells11152336 35954181 PMC9367584

[6] Sheibani N. Connective tissue growth factor: A key factor among mediators of tissue fibrosis. J Ophthalmic Vis Res (2022) 17(4):449–52. doi: 10.18502/jovr.v17i4.12294 PMC9806321 36620701

[7] Kramann R Schneider RK DiRocco DP MaChado F Fleig S Bondzie PA. Perivascular Gli1+ progenitors are key contributors to injury-induced organ fibrosis. Cell Stem Cell (2015) 16(1):51–66. doi: 10.1016/j.stem.2014.11.004 25465115 PMC4289444

[8] Luo X Yang S Liang J Zhai Y Shen M Sun J. Choroidal pericytes promote subretinal fibrosis after experimental photocoagulation. Dis Models Mech (2018) 11(4):dmm032060. doi: 10.1242/dmm.032060 PMC5963858 29622551

[9] Corano Scheri K Lavine JA Tedeschi T Thomson BR Fawzi AA. Single-cell transcriptomics analysis of proliferative diabetic retinopathy fibrovascular membranes reveals AEBP1 as fibrogenesis modulator. JCI Insight (2023) 8(23):e172062. doi: 10.1172/jci.insight.172062 37917183 PMC10896003

[10] Farnoodian M Halbach C Slinger C Pattnaik BR Sorenson CM Sheibani N. High glucose promotes the migration of retinal pigment epithelial cells through increased oxidative stress and PEDF expression. Am J Physiol Cell Physiol (2016) 311(3):C418–36. doi: 10.1152/ajpcell.00001.2016 PMC5129761 27440660

[11] Zhao Z Zhang Y Zhang C Zhang J Luo X Qiu Q. TGF-β promotes pericyte-myofibroblast transition in subretinal fibrosis through the Smad2/3 and Akt/mTOR pathways. Exp Mol Med (2022) 54(5):673–84. doi: 10.1038/s12276-022-00778-0 PMC9166792 35624154

[12] Soliman H Tung LW Rossi FMV. Fibroblast and myofibroblast subtypes: single cell sequencing. Methods Mol Biol (2021) 2299:49–84. doi: 10.1007/978-1-0716-1382-5_4 34028734

[13] Xie T Wang Y Deng N Huang G Taghavifar F Geng Y. Single-cell deconvolution of fibroblast heterogeneity in mouse pulmonary fibrosis. Cell Rep (2018) 22(13):3625–40. doi: 10.1016/j.celrep.2018.03.010 PMC5908225 29590628

